# GraphRAG-Enabled Local Large Language Model for Gestational Diabetes Mellitus: Development of a Proof-of-Concept

**DOI:** 10.2196/76454

**Published:** 2026-01-05

**Authors:** Edmund Evangelista, Fathima Ruba, Salman Bukhari, Amril Nazir, Ravishankar Sharma

**Affiliations:** 1 College of Technological Innovation, Zayed University, Abu Dhabi, United Arab Emirates, 971 25993761; 2 Department of Computer Science and Artificial Intelligence, Plaksha University, Chandigarh, India; 3 Department of Electrical Engineering, Capital University of Science and Technology, Islamabad, Pakistan

**Keywords:** artificial intelligence for health care, generative AI, knowledge graph, retrieval augmented generation, large language model, gestational diabetes mellitus, explainable AI in medicine, GDM, artificial intelligence

## Abstract

**Background:**

Gestational diabetes mellitus (GDM) is a prevalent chronic condition that affects maternal and fetal health outcomes worldwide, increasingly in underserved populations. While generative artificial intelligence (AI) and large language models (LLMs) have shown promise in health care, their application in GDM management remains underexplored.

**Objective:**

This study aimed to investigate whether retrieval-augmented generation techniques, when combined with knowledge graphs (KGs), could improve the contextual relevance and accuracy of AI-driven clinical decision support. For this, we developed and validated a graph-based retrieval-augmented generation (GraphRAG)–enabled local LLM as a clinical support tool for GDM management, assessing its performance against open-source LLM tools.

**Methods:**

A prototype clinical AI assistant was developed using a GraphRAG constructed from 1212 peer-reviewed research articles on GDM interventions, retrieved from the Semantic Scholar API (2000‐2024). The GraphRAG prototype integrated entity extraction, KG construction using Neo4j, and retrieval-augmented response generation. The performance was evaluated in a simulated environment using clinical and layperson prompts, comparing the outputs of the systems against ChatGPT (OpenAI), Claude (Anthropic), and BioMistral models across 5 common natural language generation metrics.

**Results:**

The GraphRAG-enabled local LLM showed higher accuracy in generating clinically relevant responses. It achieved a bilingual evaluation understudy score of 0.99, Jaccard similarity of 0.98, and BERTScore of 0.98, outperforming the benchmark LLMs. The prototype also produced accurate, evidence-based recommendations for clinicians and patients, demonstrating its feasibility as a clinical support tool.

**Conclusions:**

GraphRAG-enabled local LLMs show much potential for improving personalized GDM care by integrating domain-specific evidence and contextual retrieval. Our prototype proof-of-concept serves two purposes: (1) the local LLM architecture gives practitioners from underserved locations access to state-of-the-art medical research in the treatment of chronic conditions and (2) the KG schema may be feasibly built on peer-reviewed, indexed publications, devoid of hallucinations and contextualized with patient data. We conclude that advanced AI techniques such as KGs, retrieval-augmented generation, and local LLMs improve GDM management decisions and other similar conditions and advance equitable health care delivery in resource-constrained health care environments.

## Introduction

The growing use of electronic medical records linking diverse patient characteristics and prescription choices with positive treatment outcomes in large-scale use cases has resulted in platforms that guide optimal treatment options. For example, Sharma et al [1] presented an approach for delivering personalized health care as a means of effectively using scarce medical resources in underserved regions and populations, supporting the value of artificial intelligence (AI)–driven systems in such settings. While machine learning (ML) and data analytics have generated individualized treatment recommendations for improving outcomes, “these works focused on making broad [largely drug class level] treatment recommendations independently of specific drug and dose considerations... [whereas] guidelines and landmark trials highlight important drug- and dose-dependent variations in treatment efficacy, safety, and risk profiles” [2]. In short, personalized medicine should account for contextual variations in seeking more effective, cost-efficient treatments with better outcomes. This study presents an approach to clinical support to time- and resource-constrained practitioners using a generative artificial intelligence (GenAI) approach to treat a serious medical condition afflicting young mothers and their children with increasing alacrity. Such a need is particularly acute in the socioeconomically disadvantaged regions of the world.

**Figure 1.**
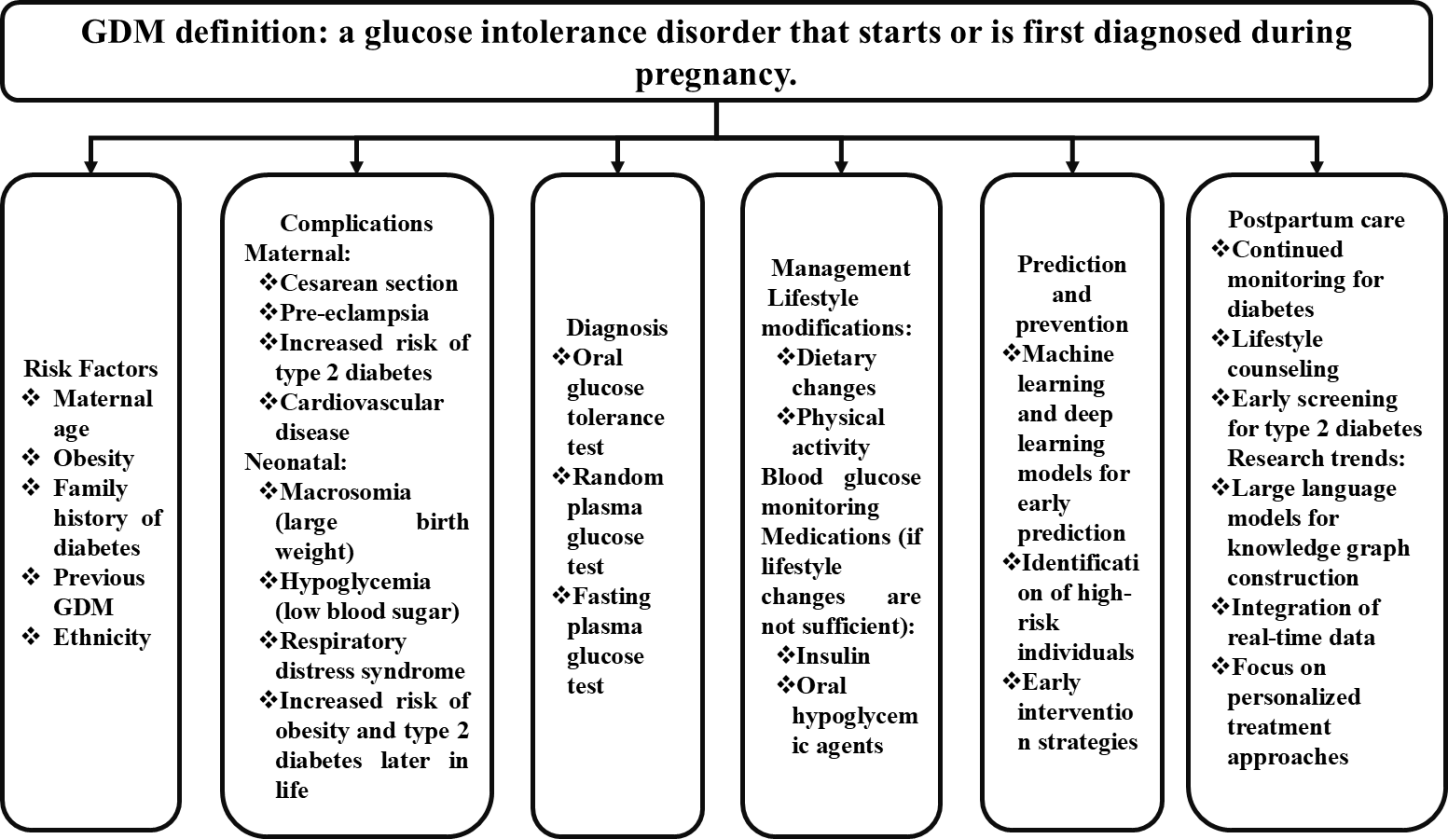
Medical characterization of gestational diabetes mellitus. GDM: gestational diabetes mellitus.

Gestational diabetes mellitus (GDM) is a significant global health concern affecting many pregnancies [3]. Defined as glucose metabolism imbalance first detected during pregnancy, the International Association of Diabetes in Pregnancy Study Group reports that “GDM is not only related to perinatal morbidity but also to an increased risk of diabetes and cardiovascular disease in the mother in later life, and childhood obesity in the offspring” [4]. The pooled global prevalence was 14% in 2021, with the highest occurrence in the Middle East - North Africa (27.6%), Southeast Asia (20.8%), and among high-income countries (14.2%) [5]. There is considerable agreement among medical practitioners that the development of GDM could be influenced by various risk factors, including maternal age, obesity, family history of diabetes, previous occurrences of GDM, and specific ethnic backgrounds [6,7]. This is illustrated in Figure 1 (data sources: [3,8-10]) as the medical characterizations of GDM comprising factors such as diagnosis, risks, prediction, management, complications, and postpartum care.

Also, of concern to the WHO is that GDM leads to various complications for both affected mothers and their offspring, such as increased risks of cesarean delivery, pre-eclampsia, and type 2 diabetes (T2D) for mothers. Children are at higher risk of macrosomia, hypoglycemia, respiratory distress syndrome, and an increased likelihood of developing obesity and T2D later in life [11]. The long-term health risks include elevated chances of developing T2D and cardiovascular diseases for both mother and child [12]. In the Global South and developing countries [8,13], GDM presents significant challenges due to:

1. Higher prevalence rates in certain regions, particularly South Asia and the Middle East.
2. Limited health care resources for screening, diagnosis, and management.
3. Genetic factors in certain ethnic groups increase GDM risk.
4. Rapid urbanization and lifestyle changes leading to increased obesity rates.
5. Potential underdiagnoses due to lack of routine screening.

Effective GDM treatment requires multiple diagnostic tests, including oral glucose tolerance tests, random plasma glucose tests, and fasting plasma glucose tests. The treatment options include regular blood glucose monitoring, dietary modifications, lifestyle changes, and, when necessary, pharmacological interventions such as insulin or oral hypoglycemic agents [9]. The recent advancements in AI-driven tools, such as the AI Drug Mix and Dose Advisor developed for T2D [2], have shown potential in optimizing pharmacological interventions by customizing drug and dose recommendations to individual patient profiles. Similar approaches could be valuable in improving glycemic management in GDM cases, enhancing personalized care in postpartum treatment, drug discovery with therapy, and reducing long-term risks of developing chronic diseases in general.

Despite growing interest in AI-driven clinical support, current models often struggle to integrate diverse, multisource medical data into actionable insights, especially in conditions such as GDM, where missing information and diagnostic delays contribute to less desirable outcomes. These limitations are particularly prominent in resource-constrained settings, where systemic challenges, such as insufficient screening tools, lack of standardized care protocols, and limited provider training, complicate effective diagnosis and treatment [8,13]. As a result, the timely and effective treatment of GDM remains difficult, further endangering maternal and fetal health.

In such contexts, the unavailability of specialized professionals, economic constraints, and cultural challenges also influence treatment adherence and engagement [14,15]. The limited awareness between both the public and health care providers continues to contribute to improper management of GDM [16], reinforcing the urgent need for robust, context-sensitive clinical decision support [17,18].

To address these gaps, we propose a novel solution using specialized GenAI techniques for GDM management. Specifically, we develop a proof-of-concept (PoC) of a clinical support system that uses a knowledge graph (KG) supporting a local large language model (LLM). This system extracts and integrates intervention strategies from peer-reviewed research to support physicians in making contextually relevant treatment decisions.

Standalone local LLMs, however, face known limitations, including hallucinations and reduced reliability when handling domain-specific, complex queries [19]. To address these issues, we introduce a retrieval-augmented generation (RAG) mechanism that improves the accuracy and relevance of outputs by supplementing the LLM with contextual data [20,21]. This hybrid approach could elevate the clinical utility of GenAI for complex, low-resource health care scenarios such as GDM.

By generating structured, evidence-informed recommendations in real time, our system lays the foundation for scalable and explainable AI support tools customized to maternal health. The following section reviews previous ML and LLM-based approaches to GDM detection and prediction, positioning our work within this evolving research landscape. It is stated at this juncture that while the distinction between LLMs and local LLMs is clear, it is less so between local LLMs and small language models (SLMs). The prototype developed in this study assumed a local LLM architecture but could be repurposed as SLMs, particularly in resource-constrained locations of the Global South. A concise feature comparison of LLMs, local LLMs, and SLMs is provided in Textbox 1.

Textbox 1. **Feature comparison of large language models, local large language models, and small language models.**

**Large language models**

Large language models (LLMs) are typically based on deep learning, trained on massive amounts of text and increasingly multimedia data to understand, generate, and manipulate human language. LLMs work by learning to predict the next word in a sequence based on the context of the input prompt, using billions of parameters to refine these predictions. They excel at natural language processing tasks such as text completion, translation, summarization, question-answering, and content generation.

**Local LLMs**

Local LLMs run inside the private data center of an entity or organization. Local LLMs are fine-tuned with the organization’s data (eg, patient records or standard rules) and can provide specific context to a query or prompt that general-purpose chatbots cannot or should be legally allowed to deliver. Particularly in the domains of sensitive and confidential data (such as a patient’s medical conditions), such prompts may have to be subject to rigorous access, authentication, and accounting controls.

**Small language model**

A small language model is designed to understand and generate natural language, similar to LLMs, but on a much smaller scale, with fewer parameters and a simpler architecture. Small language models are optimized for efficiency and can be deployed on resource-constrained devices like smartphones or local servers, offering benefits such as faster training and execution, lower energy consumption, and improved privacy by allowing for on-device processing and less reliance on cloud connectivity. A use case could be first responders in emergency room situations.

Recent advances in ML have shown promise in improving the early diagnosis and personalized management of chronic conditions such as GDM. These models identify high-risk individuals during pregnancy, customize treatment plans, and ultimately enhance maternal and neonatal health outcomes. Several studies have developed ML algorithms that account for demographic variations, for example [22,23], present models customized to Asian women [10] used decision trees and ensemble learning for early GDM detection, reporting high sensitivity and specificity. However, these models often fail to capture the full complexity of GDM-related factors.

The efforts to improve model interpretability include research, such as meta-reviews of clinical studies on complications during pregnancy and their treatments [24], on clinically explainable ML approaches for blood glucose monitoring [25,26], and the use of extreme gradient boosting to identify key risk factors [27]. However, several studies [25,26,28,29] note limitations in integrating high-quality datasets, supporting real-time interventions, or embedding models within clinical systems. Table 1 presents these representative models, underscoring the trade-offs between accuracy, interpretability, and practical usability.

**Table 1.** Representative research deep learning or machine learning models for predicting gestational diabetes mellitus.

Study	Year	Model	Key contributions and limitations
Kokori et al [22] and Kumar et al [23]	2024	Demographic-specific ML^a^ model	KCs^b^: Accurate predictions for specific demographics (Asian women). Limits: Limited integration into health care systems.
Kurt et al [10]	2023	Decision trees and ensemble	KCs: High sensitivity and specificity. Limits: Fails to capture all GDM^c^-related factors.
Wu et al [29]	2024	Clinically interpretable ML	KCs: Emphasized interpretable models for GDM. Limits: Limited real-time application.
Wu et al [25]	2022	ML-based models	KCs: Importance of high-quality datasets. Limits: Lacks interpretability and integration.

a ML: machine learning.

b KC: key contribution.

c GDM: gestational diabetes mellitus.

These limitations highlight the need for models that go beyond static risk prediction to support context-aware clinical decision-making. In this regard, LLMs offer transformative potential as they generate patient-specific recommendations by synthesizing heterogeneous clinical data. When augmented with retrieval techniques, such models become more effective.

Several recent studies have discussed the expanding role of LLMs across health care domains[30]. For example, an AI system developed for liver diseases [31] provided personalized treatment strategies that improved diagnostic outcomes. Graph-based retrieval-augmented generation (GraphRAG) integration has shown benefits in nephrology by increasing output precision and reliability [20], while LLMs have supported psychotherapy automation [32] and administrative workload reduction in personalized medicine [33]. Some of these use cases are captured in Table 2, reinforcing the applicability of RAG-augmented LLMs in clinical practice.

**Table 2.** Representative use cases of artificial intelligence in clinical health care.

Study	Year	Model	Key contributions
Ge et al [31]	2024	AI^a^ model for liver diseases	Enhanced diagnostic accuracy and patient management tailored for liver diseases.
Ong et al [34]	2023	Clinical decision support system	Improved clinical decision-making with RAG^b^-enhanced LLMs^c^, offering precise predictions and treatments.
Miao et al [20]	2024	LLM-RAG for nephrology	Improved accuracy and reliability in nephrology advice by integrating RAG with LLMs.
Stade et al [32]	2024	LLMs in psychotherapy	Explored the potential of LLMs to support and potentially automate aspects of psychotherapy.
Tripathi et al [33]	2024	Personalized medicine AI model	Demonstrated how LLMs can automate administrative tasks, reducing clinicians’ workload from electronic medical records.

a AI: artificial intelligence.

b RAG: retrieval-augmented generation.

c LLM: large language model.

Noting the above, this paper proposes a novel architecture for GDM care that integrates (1) a *local LLM* for domain-specific control and privacy, (2) an *RAG* engine for contextual grounding, and (3) a *domain-specific KG* to capture interrelated medical evidence.

This combination enables real-time generation of explainable, evidence-informed treatment recommendations for GDM management, even in resource-constrained settings. As compared with previous studies, such as those by Nambiar et al [2] and Tripathi et al [33], which focused on general dosing automation or task simplification, this study addresses a critical gap: the need for adaptive, fine-grained, and explainable intervention support in the prenatal context.

From a technical standpoint, our contributions are (1) the construction of a GDM-specific KG derived from peer-reviewed literature; (2) the use of RAG-enhanced local-LLMs to retrieve, contextualize, and generate targeted care pathways; and (3) a PoC system architecture that is interpretable, domain-grounded, and designed for offline, privacy-preserving environments.

The PoC will support timely intervention and align with the practical realities of underserved clinical contexts; consider the plight of a rural doctor in the Global South, where internet connectivity, specialist clinician availability, and cutting-edge expertise may be limited. It represents a step toward deploying technically robust and clinically meaningful AI to applications of acute need.

Following this introduction, the remainder of this paper is organized as follows. The next section addresses the methods, and specifically, a description of developing design artifacts for a PoC. In the Results section, we put the system through simulated scenarios and test the responses for accuracy, bias, and performance benchmarking. In the Discussion section, we present the principal findings along with an analysis of key contributions of the research. The paper ends with a section on Conclusions, which also covers limitations and suggestions for further research.

## Methods

### Prototyping a PoC

Health care professionals, particularly those in densely populated and resource-constrained regions of the Global South, often face significant challenges in accessing timely, evidence-based medical insights. Attending training sessions or reviewing vast volumes of literature under time pressure is impractical, especially in scenarios where specialist expertise or standardized guidelines are lacking. Our approach uses computational methods to extract, structure, and contextualize medical knowledge using GenAI and KG technologies to address this need.

**Figure 2.**
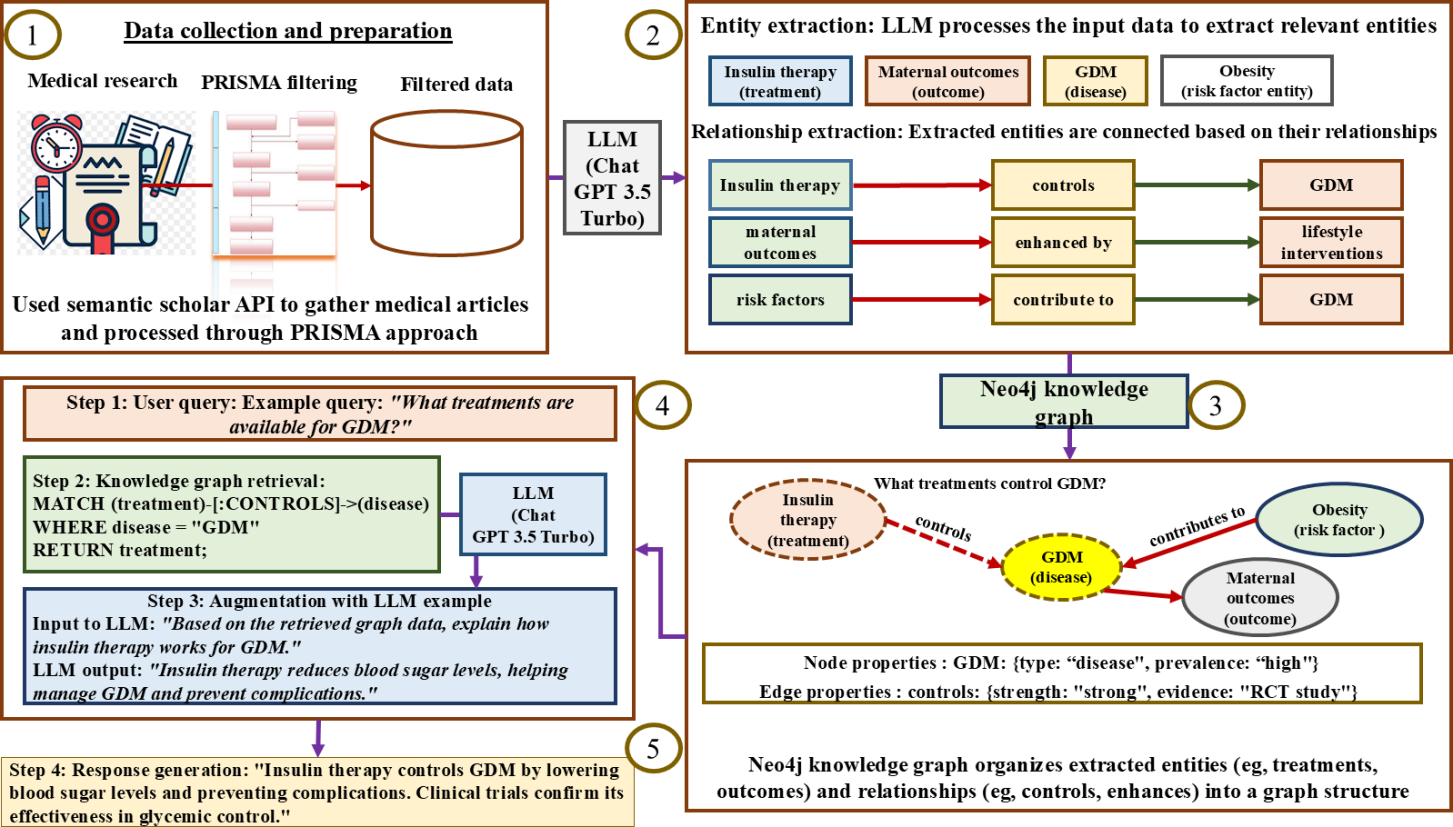
Process flow of the proposed graph-based retrieval-augmented generation approach, showing data collection, entity extraction, knowledge graph construction, and retrieval-augmented generation for AI-assisted clinical support for gestational diabetes mellitus. API: application programming interface; GDM: gestational diabetes mellitus; LLM: large language model; PRISMA: Preferred Reporting Items for Systematic Reviews and Meta-Analyses.

Our primary objective was to develop a PoC of a clinical AI assistant that would support the management of GDM. This GraphRAG-based architecture combines entity extraction from published research, KG construction, and RAG to generate clinically grounded, context-aware responses. As illustrated in Figure 2, the PoC framework follows a 5-stage pipeline.

- Data collection and preparation: The Semantic Scholar API retrieved relevant research articles on GDM interventions. A PRISMA (Preferred Reporting Items for Systematic Reviews and Meta-Analyses)–guided filtering process was applied to ensure that inclusion criteria were met, resulting in a refined corpus of 1212 high-quality articles.
- Entity extraction: Using GPT-3.5 Turbo (OpenAI) and few-shot prompting, entities such as treatments, outcomes, risk factors, and disease indicators were extracted from full-text articles. Semantic consolidation (eg, grouping “low-carb diet” and “reduced carbohydrate intake”) ensured terminological consistency.
- KG construction: Extracted entities and their relationships were encoded into a Neo4j graph database. The graph allowed efficient traversal of clinical pathways, such as connecting interventions to outcomes and risk profiles. Each node and edge pair was annotated with medical metadata, such as intervention strength, evidence level, or prevalence.
- Query processing and graph retrieval: When a user query is submitted (eg, “What treatments control GDM?”), the system was designed to retrieve relevant subgraphs using Cypher queries. These results are then passed to the LLM for augmentation and contextual response generation by incorporating patient records.
- Response generation: The final output is a clinically coherent and relevant response integrating retrieved evidence and a generative explanation. For example, based on retrieved data, the model might respond: “Insulin therapy controls GDM by lowering blood sugar levels and preventing complications.” If asked why, the system might explain: “Insulin enables glucose uptake by cells throughout the body, particularly muscle and fat cells, by facilitating glucose transport across cell membranes. Without adequate insulin, glucose accumulates in the bloodstream while cells are starved of this essential energy source.”

This multistep process would allow the system to access reputable and current medical research to produce explainable, evidence-grounded outputs for clinical decision support. Each component of this workflow is further detailed in the following subsections.

### Data Collection

**Figure 3.**
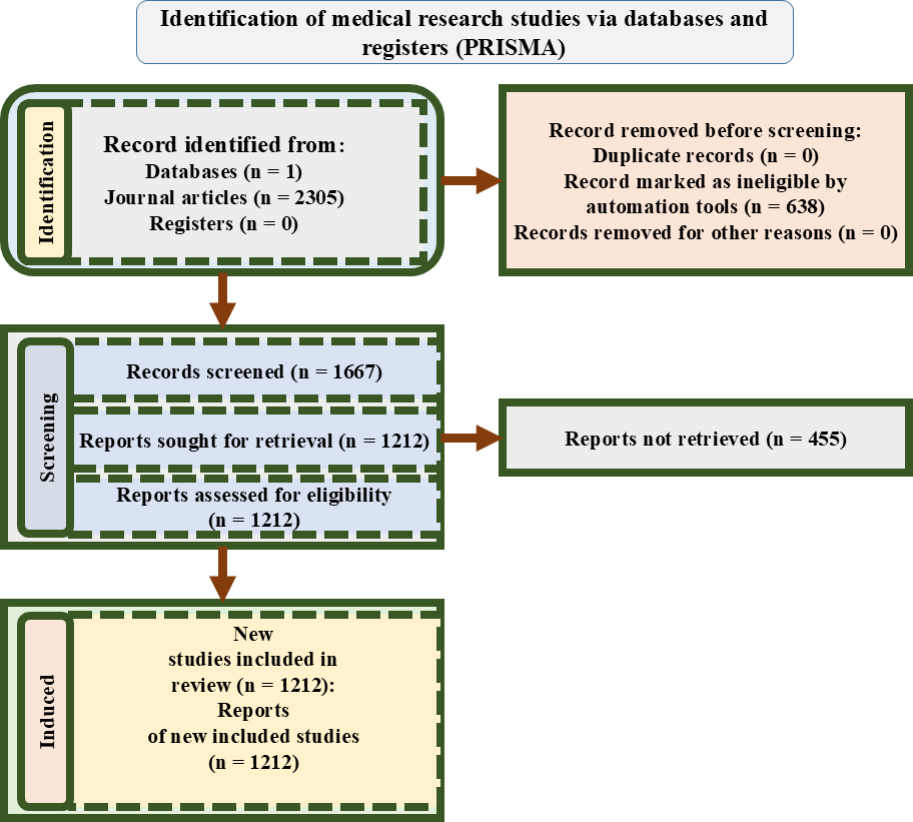
PRISMA flow diagram showing the systematic data collection and filtering process, detailing identification, screening, eligibility assessment, and inclusion of research articles for knowledge graph construction.

To develop a high-quality domain-specific KG for GDM, we conducted a systematic search using the Semantic Scholar API [35], a widely used biomedical research platform. The query term “gestational diabetes interventions” was selected to target studies focused on treatment strategies and clinical outcomes. The search was restricted to articles published between January 2000 and May 2024, to cover both foundational and contemporary research. The data collection and filtering process adopted PRISMA guidelines, as illustrated in Figure 3.

- Identification: The initial search produced 2305 journal articles. No records were found from registers. Automated filters removed 638 ineligible records based on metadata mismatches or irrelevant domains. No duplicate entries were detected.
- Screening: The remaining 1667 articles were screened by 2 reviewers (FR and SB) based on titles and abstracts. This stage ensured that only articles related to GDM diagnosis, treatment, management, or intervention outcomes were retained.
- Eligibility: A total of 1212 full-text articles were deemed eligible based on the inclusion criteria. Articles were excluded at this stage (n=455) due to full-text unavailability, access limitations, or insufficient clinical relevance.
- Inclusion: The final corpus consisted of 1212 peer-reviewed studies, all of which were used to extract entities and construct the GDM-focused KG.

While Semantic Scholar provided comprehensive coverage and metadata-rich access, reliance on a single source introduces potential limitations, such as limited representation of non-English or region-specific research and sensitivity to keyword variations. Future work could explore multilingual database integration and broader query strategies to reduce potential selection bias.

Nonetheless, for developing our PoC, the selected dataset offered sufficient diversity and clinical validity to enable meaningful experimentation and system development.

### Entity Extraction

Following the curation of the GDM research corpus, the next step involved extracting clinically relevant concepts, including treatments, risk factors, and outcomes, from the published research. This process was executed using OpenAI’s GPT-3.5 Turbo 16K API [36], which supported advanced natural language processing for domain-specific knowledge extraction. Rather than relying on pretrained biomedical ontologies, we adopted a lightweight prompting-based approach aligned with our PoC’s experimental and modular goals.

A few-shot prompting strategy was applied to guide the language model in identifying and structuring entities of interest in a usable format. Guided by 3 medical doctors, the prompts were manually engineered to show expected outputs, such as intervention types (eg, insulin therapy, diet, and physical activity), intervention parameters (eg, frequency, duration, and dosage), and associated maternal and infant outcomes. This enabled the model to consolidate synonymous or semantically related expressions (such as “low carbohydrate diet” and “reduced carb intake”) into a unified entity representation. The same prompts also encouraged disambiguation of overlapping terms and discouraged the duplication of entities across articles.

The outputs were parsed into structured formats, which included both individual entities and the semantic relationships among them, for example, linking “insulin therapy” as a treatment that “controls” GDM, or connecting “smartphone-based lifestyle interventions” to enhanced “maternal outcomes.” These entities and their connections were then directly integrated into the KG in the next stage of development.

This stage of entity extraction was led by the coauthor (FR), who specializes in bioinformatics and uses a technique we describe as “medical prompt engineering.” The objective was to simulate how future clinical AI assistants might extract structured knowledge from unstructured medical literature autonomously. However, we acknowledge that such extractions would require validation by specialist health care professionals to ensure accuracy and reliability for clinical deployment.

**Figure 4.**
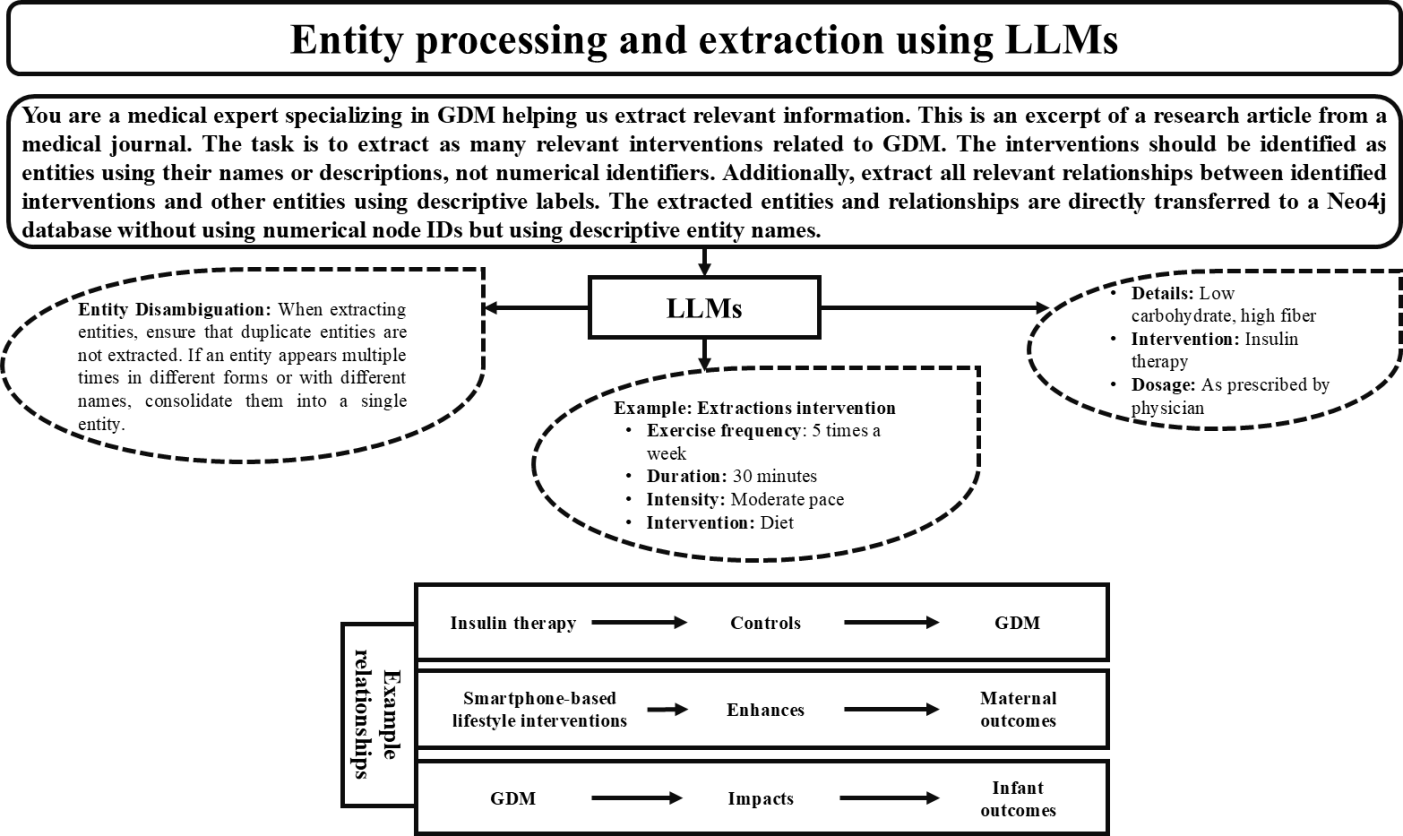
Entity extraction workflow using large language models. The diagram is an example of the process for extracting interventions, risk factors, and relationships, which produces structured and context-aware knowledge representation for gestational diabetes mellitus management. GDM: gestational diabetes mellitus; LLM: large language model.

The overall entity extraction workflow, including prompt design, model guidance, semantic structuring, and preparation for graph integration, is visualized in Figure 4.

### Construction of the KG

Upon completion of the entity and relationship extraction, the structured data were integrated into a KG using Neo4j, a widely used open-source graph database [37]. Neo4j is optimized for representing interconnected biomedical data, making it well-suited for capturing the multifactorial nature of GDM management, which involves dynamic relationships between interventions, risk factors, outcomes, and complications [38].

The KG construction process involved linking each extracted entity, such as insulin therapy, dietary strategies, or risk factors like obesity, to its semantically relevant mappings using directional edges labeled with relationship types (eg, “controls,” “contributes to,” and “enhances”). Each node was annotated with descriptive labels and properties derived from the literature, and relationships were encoded with metadata such as source references or study types, when available.

All nodes and edges were imported into Neo4j through a structured ingestion pipeline, enabling clinicians or researchers to query the KG using the Cypher query language. This functionality allowed for exploratory clinical queries, such as identifying interventions most frequently associated with improved maternal outcomes in high-risk GDM cases or tracing evidence paths for specific treatment combinations.

**Figure 5.**
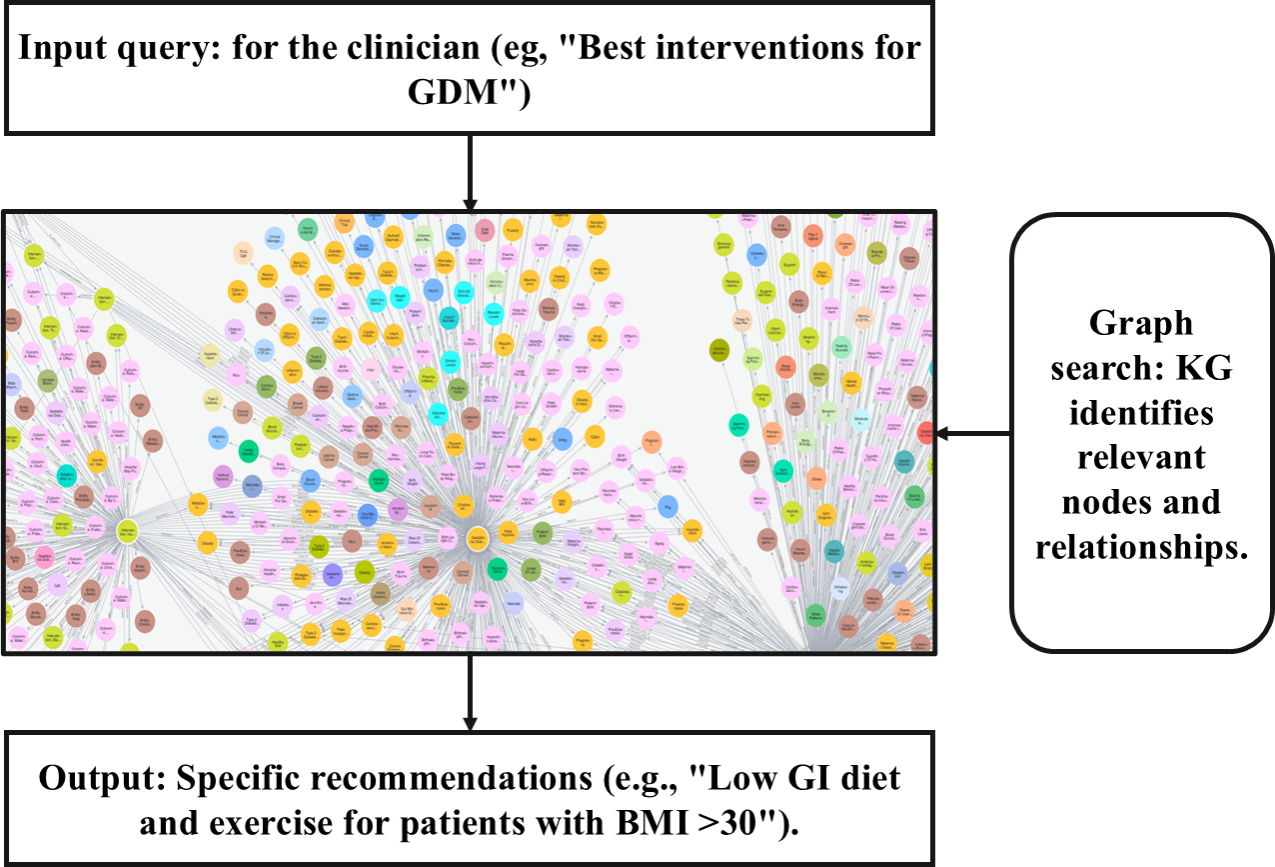
Knowledge graph–powered clinical support system for gestational diabetes mellitus. The graph-based search retrieves relevant interventions and relationships, giving treatment recommendations. GDM: gestational diabetes mellitus; KG: knowledge graph.

The resulting KG facilitated context-aware clinical decision support by surfacing specific evidence-informed insights. For example, a clinician’s query, such as “What are the best interventions for GDM in patients with a BMI over 30?” could retrieve targeted graph segments linking relevant interventions (eg, low glycemic index diet and structured exercise regimens) to outcomes validated in the literature. This dynamic capability is depicted in Figure 5, which illustrates a representative graph traversal initiated by a clinician’s question, leading to personalized treatment recommendations based on the structural relationships captured in the KG.

The KG serves as the core reasoning backbone of the prototype clinical assistant, consolidating distributed medical evidence into a queryable visual knowledge substrate that can be updated as new medical evidence emerges.

### KG-Based RAG

To enhance the clinical utility of the constructed KG, we then implemented an RAG approach [39]. This hybrid architecture combines traditional retrieval mechanisms with generative LLMs to produce contextually grounded and medically sound responses. In clinical settings, where decision-making depends on subtle interpretation and evidence-based insights, this integration mitigates the limitations of standalone generative systems like SLMs.

While LLMs, such as ChatGPT (OpenAI), can produce fluent and context-aware responses, they are prone to hallucinations, outdated knowledge, and domain-specific inaccuracies [19]. Conversely, RAG addresses these gaps by coupling LLMs with reputable (peer-reviewed) external knowledge sources. For example, no medical claim, such as bleach being a valid treatment for COVID-19, would have gone into the KG. In our PoC, entity-aware retrieval from the Neo4j-based GDM KG provides factual context, which the LLM then uses to generate a tailored response. This integration significantly improves factual grounding and interpretability, essential in critical domains, such as maternal health [20,21].

**Figure 6.**
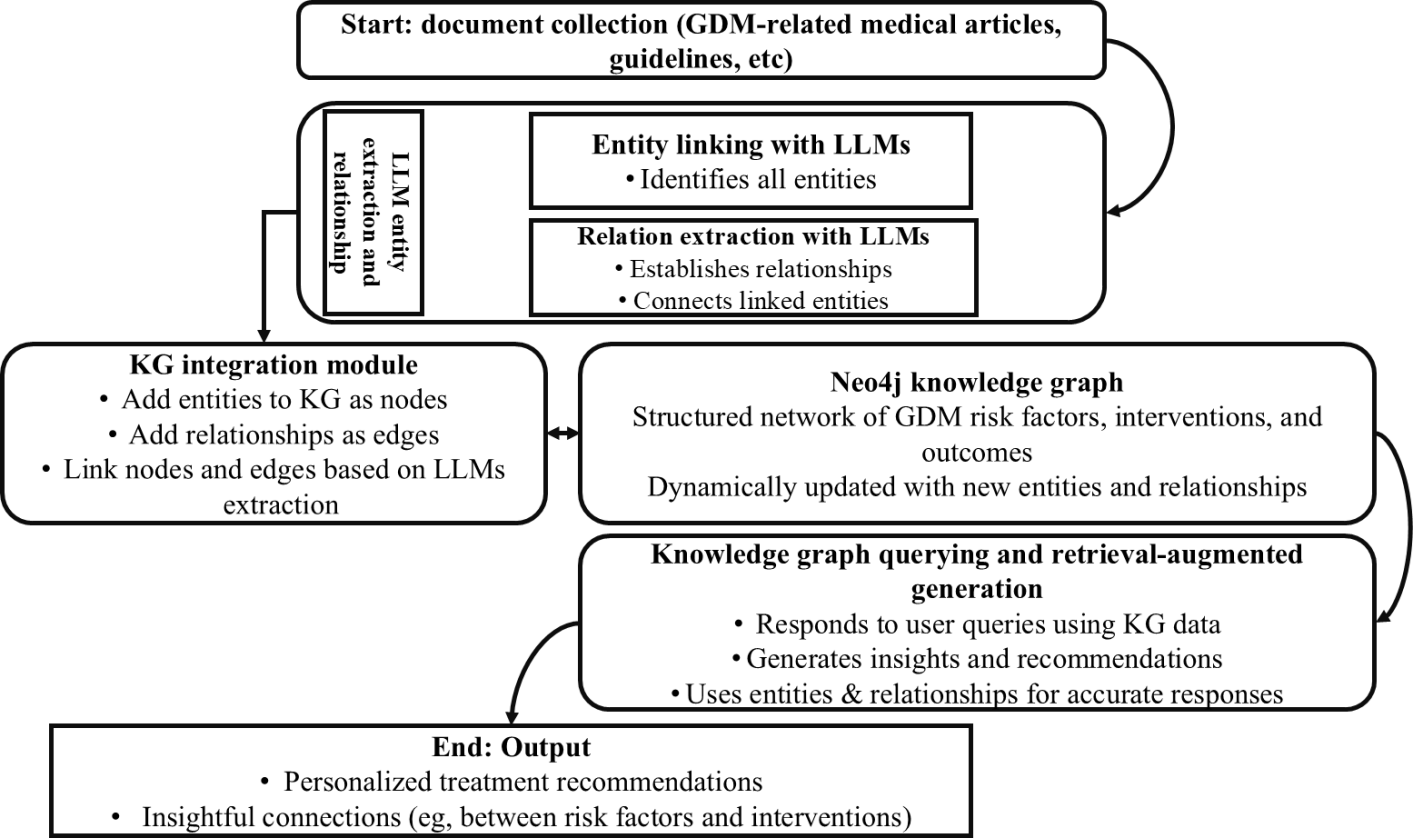
End-to-end process flow of the graph-based retrieval-augmented generation solution. The pipeline processes medical literature and patient data, integrating them into a structured knowledge graph for AI-driven clinical decision support. GDM: gestational diabetes mellitus; KG: knowledge graph; LLM: large language model.

**Figure 7.**
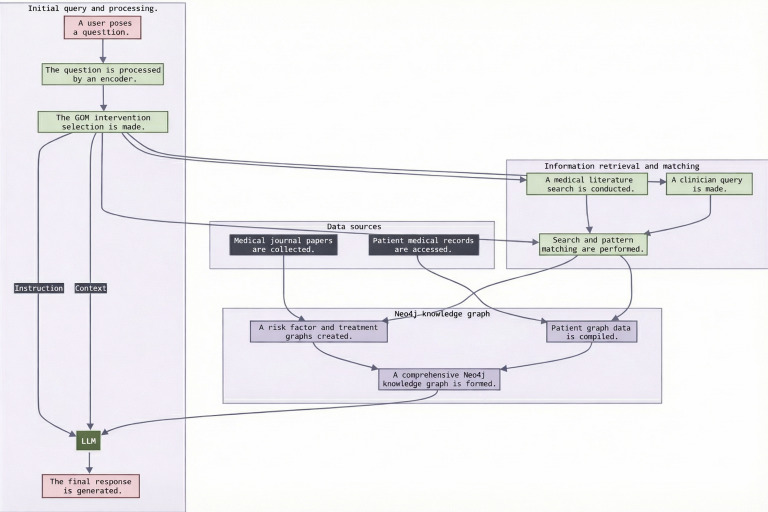
Structured retrieval and response generation process in graph-based retrieval-augmented generation. The diagram shows how clinician queries interact with medical knowledge sources, pattern matching, and graph-based retrieval to enhance artificial intelligence–generated responses. GDM: gestational diabetes mellitus; LLM: large language model.

Using the PoC follows a 5-stage pipeline, visualized in Figures 6 and 7. Beginning with an initial clinical query, the system encodes the user input and dynamically retrieves semantically matched information from the KG. This process accounts for risk factors, interventions, and patient-specific context, including medical records and socioeconomic profiles, thereby aligning output with real-world variability in treatment planning.

Figure 6 shows the underlying LLM-KG pipeline, including entity extraction, relationship linking, and graph query generation. Figure 7 offers a complementary perspective by emphasizing end-to-end data flow, from patient query and literature matching to LLM response generation, thus highlighting how both structured (graph-based) and unstructured (textual) data are integrated to yield context-aware, personalized responses.

Although Figures 6 and 7 present a simplified overview of system functionality, the development process required iterative prompt engineering, guided tuning, and manual validation to align LLM outputs with the domain-specific vocabulary and relationships obtained from GDM research literature [32,33]. This iterative refinement helped ensure that the GraphRAG PoC consistently produces clinically meaningful recommendations rooted in the KG, avoiding spurious correlations and unverified claims.

### Evaluation Framework and Metrics

The evaluation of the GraphRAG-powered local LLM for GDM was conducted through a structured framework designed to assess both technical performance and clinical relevance. Applications of AI in health care require rigorous validation beyond prompt engineering. This study used a multidimensional evaluation process using a combination of quantitative metrics and clinician-generated prompts.

### Evaluation Objectives

The primary objective of the evaluation was to measure the effectiveness of the proposed PoC in three “fit for purpose” criteria: (1) generating clinically relevant**,** context-aware responses to queries on GDM management; (2) comparing its performance against widely used open-source LLMs in terms of accuracy and interpretability; and (3) assessing whether the retrieval-augmented approach of GraphRAG significantly improves response quality in medical decision support. These criteria reflect the critical nature of clinical decision-making, where AI-generated content’s clarity, accuracy, and contextual relevance directly affect patient safety and clinical outcomes.

### Testing Environment

The evaluation was conducted in a simulated environment, without the involvement of live patients or human participants. The GraphRAG-powered local LLM was deployed on an offline computing environment, ensuring that no external API calls or third-party cloud services influenced the test outcomes. The KG was prepopulated with medical research articles, as described in the “Prototyping a PoC” section, and served as the contextual knowledge base for all retrieval-augmented queries.

### Prompt Design and Benchmark Models

The prompts used in the evaluation were carefully crafted to simulate realistic clinical and layperson queries. These prompts were generated from two user groups: (1) *laypersons* represented by 5 contributors (the authors) simulating patient queries, verified for clarity and simplicity; and (2) *clinicians* comprising 2 general practitioners (GPs) and 1 specialist physician, who created queries based on typical clinical decision-making scenarios.

Furthermore, 2 independent medical practitioners reviewed all prompts to ensure clinical relevance (were the prompts aligned with real-world GDM management scenarios?) and content clarity (did the prompts avoid ambiguous phrasing or unrealistic edge cases?)

The GraphRAG system was then benchmarked against 3 open-source LLMs commonly used in medical AI research. The comparison is intended to analyze the performance of a domain-augmented local model (our PoC) against both general-purpose and specialized health care LLMs.

- ChatGPT [36]: A versatile, general-purpose LLM.
- Claude [40]: Known for generating coherent, contextually rich responses.
- BioMistral [41]: A domain-specific medical LLM optimized for health care contexts.

Our benchmarking compares the GraphRAG-enabled local LLM against the above 3 LLM models to assess clinical relevance, contextual accuracy, and terminological consistency. These models were selected based on availability, health care domain relevance, and ease of integration into our evaluation pipeline. While we acknowledge the increasing prevalence of open-source LLMs such as LLaMA 3 (Meta AI), due to hardware compatibility constraints and inference framework differences at the time of testing, we could not integrate LLaMA 3 within the test environment. LLaMA 3 and other emerging open-source models, such as Mistral 7B (Mistral AI) and Phi-3 (Microsoft), should be included in future benchmarking updates to expand our comparative analysis, which is suggested as future work.

### Evaluation Metrics and Rationale

Following established practices in evaluating health care AI models [42,43], we used 5 complementary metrics, each addressing a distinct dimension of AI-generated response quality. These are presented in Table 3.

**Table 3.** Metrics and their clinical significance in evaluating artificial intelligence–generated responses.

Metric	Purpose	Significance
Relevance score	Measures alignment between response content and user query.	Critical for clinical decision support, where irrelevant or off-topic answers compromise safety.
BLEU^a^ score	Evaluates syntactic similarity and phrase structure match against reference answers.	Ensures AI^b^ responses replicate validated medical language without distortion.
Jaccard similarity	Quantifies overlap in key medical terms between model response and reference.	Captures preservation of clinical terminology essential in GDM^c^ management.
BERTScore	Assesses semantic similarity using deep contextual embedding.	Evaluates whether model responses capture the intended clinical meaning beyond surface text.
METEOR	Evaluate fluency and coherence in response generation.	Ensures clarity and interpretability for both clinicians and patients.

a BLEU: bilingual evaluation understudy.

b AI: artificial intelligence.

c GDM: gestational diabetes mellitus.

Together, these metrics comprehensively address the precision, contextual relevance, and interpretability of an AI model’s outputs, which are key requirements for clinical use cases.

### Evaluation Process

The evaluation adopted the following steps:

First, each LLM, including GraphRAG, was presented with the same curated set of 20 prompts (10 from simulated layperson queries and 10 from clinicians), covering core aspects of GDM management, such as risk factors, diagnostics, treatment, and complications. The 5 coauthors (EE, FR, SB, AN, and RS) jointly drafted the layperson prompts, while clinical prompts were contributed by 2 practicing GPs and reviewed by a third medical specialist.

Second, the system’s responses were compared against reference answers, curated from clinical guidelines and expert consensus statements.

Third, evaluation was conducted in a zero-shot retrieval-augmented setting. No supervised training or fine-tuning was performed. The local LLM operated on a preconstructed KG as the contextual grounding source.

Fourth, automated evaluation metrics (bilingual evaluation understudy [BLEU], Jaccard Similarity, BERTScore, and METEOR) were computed using standard natural language processing evaluation libraries. These scores reflect surface-level accuracy, overlap in medical terminology, and semantic similarity.

Fifth, manual relevance scores were assigned by 2 independent medical reviewers on a 1‐5 scale, based on clinical applicability, specificity, and usefulness of responses.

Finally, results were averaged across all prompts and models and reported for comparative analysis in the Results section. While performance scores are high (eg, BLEU=0.99 approximately), this reflects a small, curated test set and should not be considered generalizable. CIs and interrater agreement were not calculated in this phase of the research.

### Benchmarking Scope and Qualifications

The evaluation was designed to show the technical feasibility and domain relevance of the GraphRAG framework, rather than to establish clinical deployment readiness for deployment. Consequently, the following qualifications would apply:

First, all responses were evaluated in a simulated, offline environment without involvement of human patients, real-time electronic health record data, or live clinical workflows.

Second, no supervised training or dataset splitting was involved, as the system uses RAG rather than end-to-end training. All prompts were presented statically to each LLM.

Third, as recorded in our research logs, the KG was constructed from a curated corpus of 1212 peer-reviewed, English-language articles on GDM interventions, extracted via Semantic Scholar API (2000‐2024). The KG contains approximately 2750 nodes, 5800 edges, and 18 entity types, including risk factors, therapies, dietary interventions, and outcomes.

Fourth, the evaluation prompt set, while medically validated, remains small and nonrandomized. No demographic stratification, multilingual testing, or subgroup fairness analysis was performed.

Fifth, performance metrics assessed linguistic and contextual quality only. There has been no empirical validation of clinical efficacy, patient safety, or decision-making utility.

Finally, future iterations should expand prompt diversity, compute interrater reliability scores, and explore prompt-based fairness auditing. Prospective clinical trials and feedback-integrated deployment pipelines are also planned.

### Ethical Considerations

This study involved the development and technical validation of a PoC clinical AI assistant for GDM management. The research was conducted entirely in a simulated environment without involving human participants, personal health data, or clinical interventions. Accordingly, formal ethics board approval was not required for this PoC phase of the research study.

More specifically, this was in accordance with ethical research standards for early-stage AI system development in health care. We ensured that no human participants, no personal health data, and no real-time clinical interventions resulted from this PoC phase. While fairness across subpopulations was not evaluated in this version, future efforts shall explicitly address this dimension.

### Data Source Transparency

The data used in this study were drawn exclusively from public-domain, reputable academic research, collected through the Semantic Scholar API. All articles retrieved were from peer-reviewed scientific publications, ensuring no private, sensitive, or patient-level data were accessed or processed. The use of publicly available literature aligns with ethical practices in computational biomedical research, where datasets are preferably in the public domain.

### Simulated Testing Environment

The PoC was evaluated using simulated prompts designed by the research team and reviewed by independent clinicians. No real patient interactions, medical records, or clinical environments were involved in the testing. This approach was explicitly chosen to focus on the feasibility of the proposed GraphRAG-powered knowledge retrieval and response generation approach.

All comparisons against open-source LLMs (ChatGPT, Claude, and BioMistral) were also conducted offline, with no data sent to external servers during evaluation, ensuring data security and compliance with our concern that we do not train such models with our research data.

### Responsible AI Development

The design and development of the GraphRAG framework adhered to ethical AI principles, emphasizing:

1. Transparency: Clear explanation of methods and evaluation.
2. Safety: Avoidance of deploying untested AI systems in live clinical environments.
3. Explainability: Use of a KG for contextual reasoning and improved interpretability.
4. Bias awareness: Although no patient data were used, future iterations will integrate fairness auditing to minimize algorithmic bias.

### Fairness and Demographic Representation

The development of the PoC used a small set of curated prompts authored by the research team and clinicians. Hence, no demographic, linguistic, or regional diversity was represented in the evaluation. This limitation may impact the generalizability of the system’s recommendations across patient populations. Future prototyping iterations will integrate fairness-aware evaluations**,** including prompt diversity across age, gender, geography, and language, to improve equitable performance across clinical contexts.

## Results

### System Demonstration Scenarios

The PoC beta testing in a simulated environment highlighted the feasibility of the GraphRAG-powered clinical support system for GDM management. The PoC generated personalized, clinically relevant responses to GDM-related queries, simulating interactions between patients, health care professionals, and the system.

**Figure 8.**
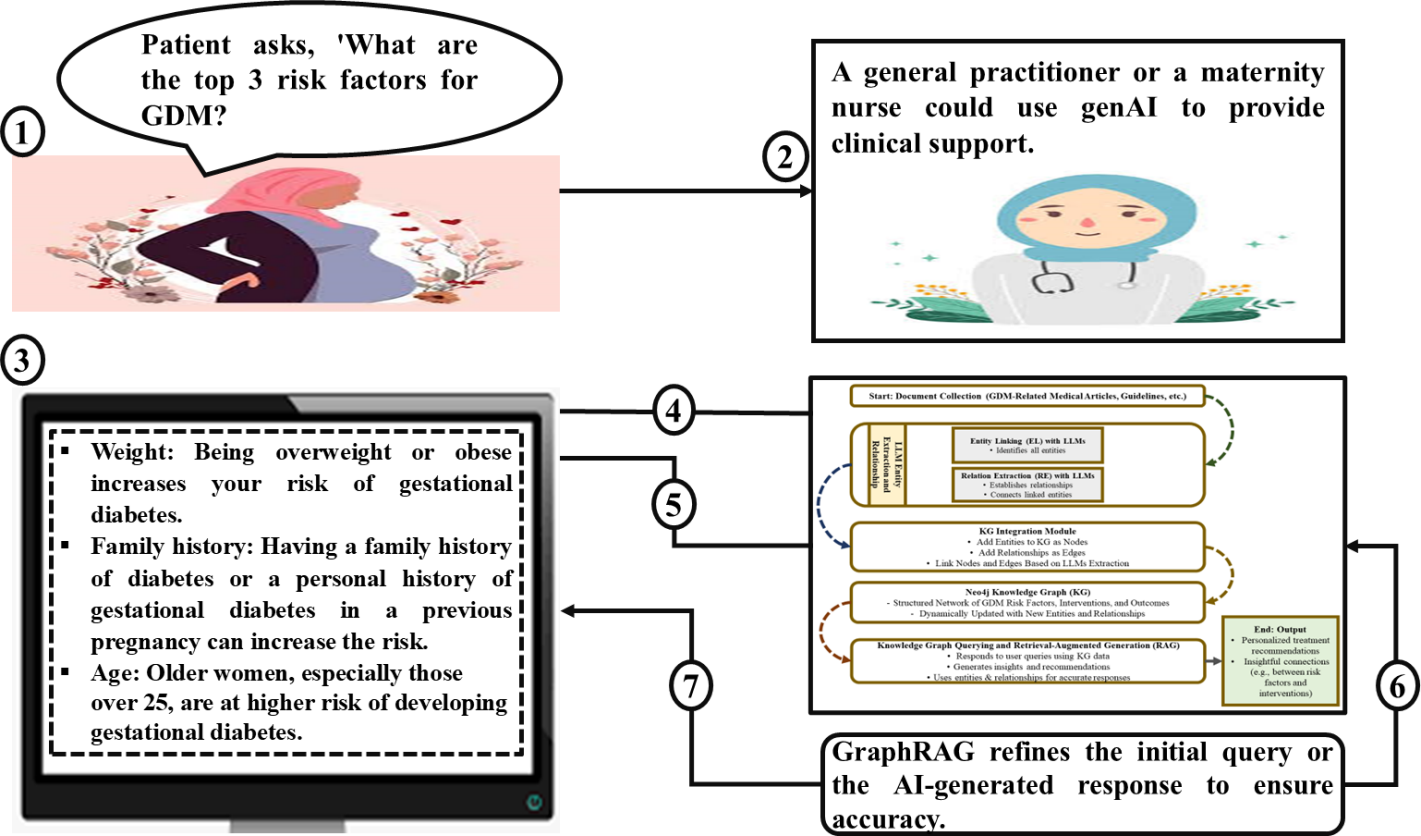
GraphRAG-based clinical support system for gestational diabetes mellitus - iconographic representation. AI: artificial intelligence; GDM: gestational diabetes mellitus; genAI: generative artificial intelligence.

**Figure 9.**
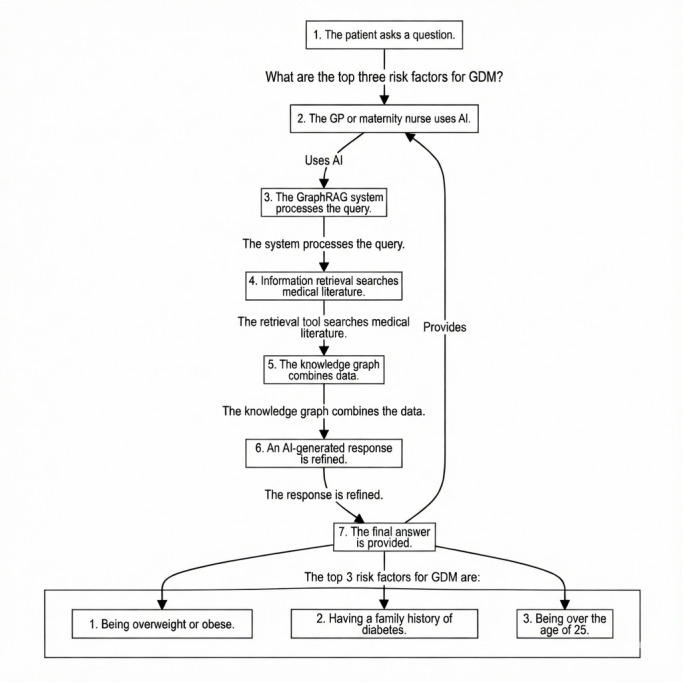
GraphRAG-based clinical support system for gestational diabetes mellitus - process flow diagram. AI: artificial intelligence; GDM: gestational diabetes mellitus; GP: general practitioner.

Figures 8 and 9 present an illustrative scenario displaying how the GraphRAG local LLM could support clinical consultations. In this example, a patient presents a question regarding the top risk factors for GDM. A health care professional, such as a GP or maternity nurse, uses the GraphRAG-enabled clinical support system to process the query into a prompt.

As illustrated in Figure 8, the system processes the initial query. It generates a concise, contextually relevant response (ie, with the benefit of the patient’s medical records), listing key GDM risk factors such as maternal weight, family history of diabetes, and maternal age. The process flow highlights how the system integrates domain-specific medical literature and patient-related contextual data through its underlying Neo4j KG, enabling it to deliver evidence-based, patient-centered recommendations.

Figure 9 further elaborates on the underlying process. The patient’s query initiates a series of steps where the system retrieves relevant interventions and relationships from the KG. The local LLM then generates a response with context-aware medical knowledge. The final advisory to the health care professional integrates the patient’s specific context and up-to-date medical research, avoiding potential inaccuracies and hallucinations.

This demonstration shows how GraphRAG can streamline clinical consultations by providing up-to-date, evidence-backed responses. Beyond basic patient interactions, the system can address more advanced clinical questions, such as identifying appropriate screening tests for GDM or evaluating intervention effectiveness. With explanation features, these capabilities support informed decision-making for health care providers and patients, enhancing the overall quality of care for GDM.

The detailed results of the system’s performance in various scenarios are presented in the following 3 subsections. The first examines the system’s responses to layperson prompts. The one that follows analyzes its performance with clinically oriented queries from 2 GPs. The third presents the comparative analysis of model performance using standard evaluation metrics across GraphRAG and 3 other LLMs.

### GraphRAG Local LLM Response to Prospective Patients’ Prompts

The GraphRAG Local-LLM generated clear, clinically relevant responses to patients’ queries related to GDM management. These prompts were designed to simulate questions that a general audience might ask during health consultations with their health care provider. The system’s responses highlighted its ability to simplify complex medical concepts while maintaining factual accuracy and clinical relevance.

Table 4 presents example prompts and the corresponding GraphRAG-generated responses. For example, when asked about the *top 3 factors determining gestational diabetes risk*, the system correctly identified key risk factors such as *weight, family history of diabetes, and maternal age*.

**Table 4.** Responses to patient prompts.

Prompt	Response
What are the top 3 factors determining gestational diabetes risk?	Weight: Being overweight or obese increases your risk of gestational diabetes. Family history: Having a family history of diabetes or a personal history of gestational diabetes in a previous pregnancy can increase the risk. Age: Older women, especially those over 25, are at higher risk of developing gestational diabetes.
How can we safeguard a baby’s health and wellness for gestational diabetes patients?	Monitor blood sugar levels, follow a healthy meal plan, engage in regular physical activity, take prescribed medications, attend prenatal check-ups, and follow healthcare guidance.
What nutrition is recommended to prevent gestational diabetes?	Eat a balanced diet high in fiber and low in added sugars and unhealthy fats. Include fruits, vegetables, whole grains, lean proteins, and healthy fats.
Are gestational diabetes patients more likely to have a cesarean or a standard delivery?	Women with gestational diabetes are more likely to have a cesarean delivery due to risks like macrosomia, shoulder dystocia, and birth trauma.
What neonatal screening should a couple with a family history of diabetes undergo before planning to have a baby? Are there precautions they could take?	Consider genetic counseling and testing. Preconception counseling can help identify risks and precautions, with personalized plans developed alongside health care professionals.

These results show the system’s capacity to convey medical information in a manner accessible to nonspecialist users. The responses accurately addressed the queries using medically appropriate language, ensuring clarity for a general audience.

### Responses to GP Prompts

The GraphRAG system was evaluated using clinically relevant prompts provided by GPs. These prompts simulated real-world clinical queries related to the diagnosis, management, and long-term planning of GDM. The system generated medically valid responses as assessed by the participating GPs and diabetologists, addressing both basic and advanced aspects of GDM care.

### Responses to Prompts From GP 1

Table 5 presents the responses generated by GraphRAG for the first set of prompts from GP 1. These prompts covered foundational aspects of GDM diagnosis and management. For example, when asked how to diagnose GDM, the system recommended the glucose challenge and tolerance tests, reflecting standard clinical guidelines. The responses addressed both diagnostic procedures and recommended clinical management steps.

The responses provided clear, evidence-aligned answers covering diagnostic tests, management strategies, fetal monitoring approaches, and long-term complications.

**Table 5.** Responses to prompts from general practitioner 1.

Prompt	Response
How do I diagnose gestational diabetes?	Gestational diabetes is typically diagnosed through a glucose challenge or glucose tolerance test. A sugary solution is consumed, and blood sugar levels are tested. If results are high, a more detailed glucose tolerance test is done. A health care provider must confirm the diagnosis and guide management.
What measures would you advise me to take once I am diagnosed with GDM^a^?	Monitor blood sugar regularly, follow a health care–guided meal plan, engage in physical activity, attend prenatal care visits, take prescribed medications, and stay in contact with your health care team.
What fetal changes should I monitor (and how) in gestational diabetes?	Monitor fetal growth via regular ultrasounds, track amniotic fluid and fetal movements (kick counts), and consult health care providers for ongoing prenatal assessments.
What are the cutting-edge medical and non-medical interventions I could take to control GDM in pregnant mothers?	Use technologies like continuous glucose monitors and personalized insulin therapy. Also, nonmedical options such as tailored diets, physical activity, and stress reduction should be applied, ideally with specialist consultation.
What are the long-term complications for GDM mothers and their offspring?	GDM increases the risk of type 2 diabetes in mothers and raises the chance of obesity, metabolic syndrome, and diabetes in children. Long-term follow-up is essential for both.

a GDM: gestational diabetes mellitus.

### Prompts From GP 2 and GraphRAG Local LLM Responses

Further evaluation was conducted using prompts from GP 2, which explored broader clinical topics, including screening tests, genetic factors, and emerging therapies. Table 6 summarizes the system’s responses.

According to the medical practitioners, the GraphRAG system generated contextually appropriate responses, addressing established clinical practices and emerging medical topics in GDM care.

**Table 6.** Responses to prompts from general practitioner 2.

Prompt	Response
What basic screening tests for GDM^a^ should I perform and monitor regularly?	Standard tests for screening and monitoring GDM include: Glucose challenge test (GCT) – a one-hour blood test after consuming a sugary drink. Oral glucose tolerance test (OGTT) – a follow-up fasting test with multiple blood draws. Glycated hemoglobin (A_1C_) – reflects average glucose levels over the past months. Regular blood glucose monitoring – daily checks using a glucometer. Consult a healthcare provider for tailored testing schedules.
On balance, does medical evidence suggest a greater genetic influence of the father or the mother for DM^b^?	Both parents can genetically influence the risk of DM. The extent varies by DM type and individual genetic profiles. Environmental factors also play a key role.
If the patient and her husband decide to go ahead with starting a family, what medical interventions can I prescribe to alleviate the risk of GDM?	Medical interventions include: Healthy diet and lifestyle habits. Regular blood glucose monitoring. Medications such as metformin, if required. Referral to a nutritionist or diabetes educator. The provider should personalize recommendations.
Can proper oral and dental care alleviate GDM during pregnancy? What are some effective measures?	There is no direct link between oral care and GDM prevention. However, good dental hygiene supports overall pregnancy health. Recommendations include brushing twice daily, flossing, and routine dental visits.
Are there promising stem-cell or epigenetic treatments that could ease my patient’s hesitance to start a family?	Stem-cell and epigenetic research show potential, but are still in the early stages. Patients should consult reproductive specialists for the latest updates and personalized advice.

a GDM: gestational diabetes mellitus.

b DM: diabetes mellitus.

### Comparative Model Performance

#### Overview of Benchmarking Procedures

The GraphRAG system was benchmarked against 3 widely used LLMs, BioMistral, ChatGPT, and Claude, using a standardized set of clinical prompts focused on GDM management. The models’ responses were evaluated using 5 quantitative metrics that assessed relevance, linguistic precision, terminology consistency, contextual understanding, and coherence.

#### Benchmarking Results

**Figure 10.**
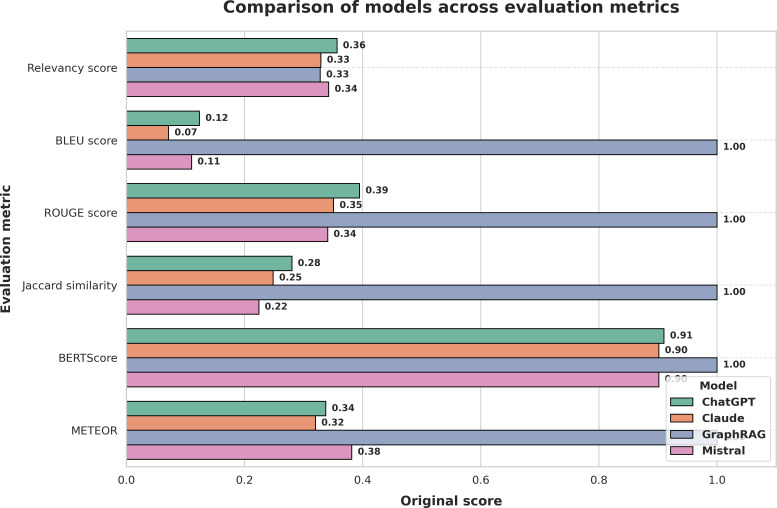
Comparative performance of GraphRAG, BioMistral, ChatGPT, and Claude across evaluation metrics. BLEU: bilingual evaluation understudy.

Figure 10 presents a comparative analysis of the models’ average performance across 5 evaluation metrics. GraphRAG achieved the highest scores in BLEU, Jaccard Similarity, and BERTScore, indicating strong alignment with clinical phrasing, preservation of key medical terms, and deep contextual accuracy. Relevance Score and METEOR also reflect competitive performance across all models.

**Figure 11.**
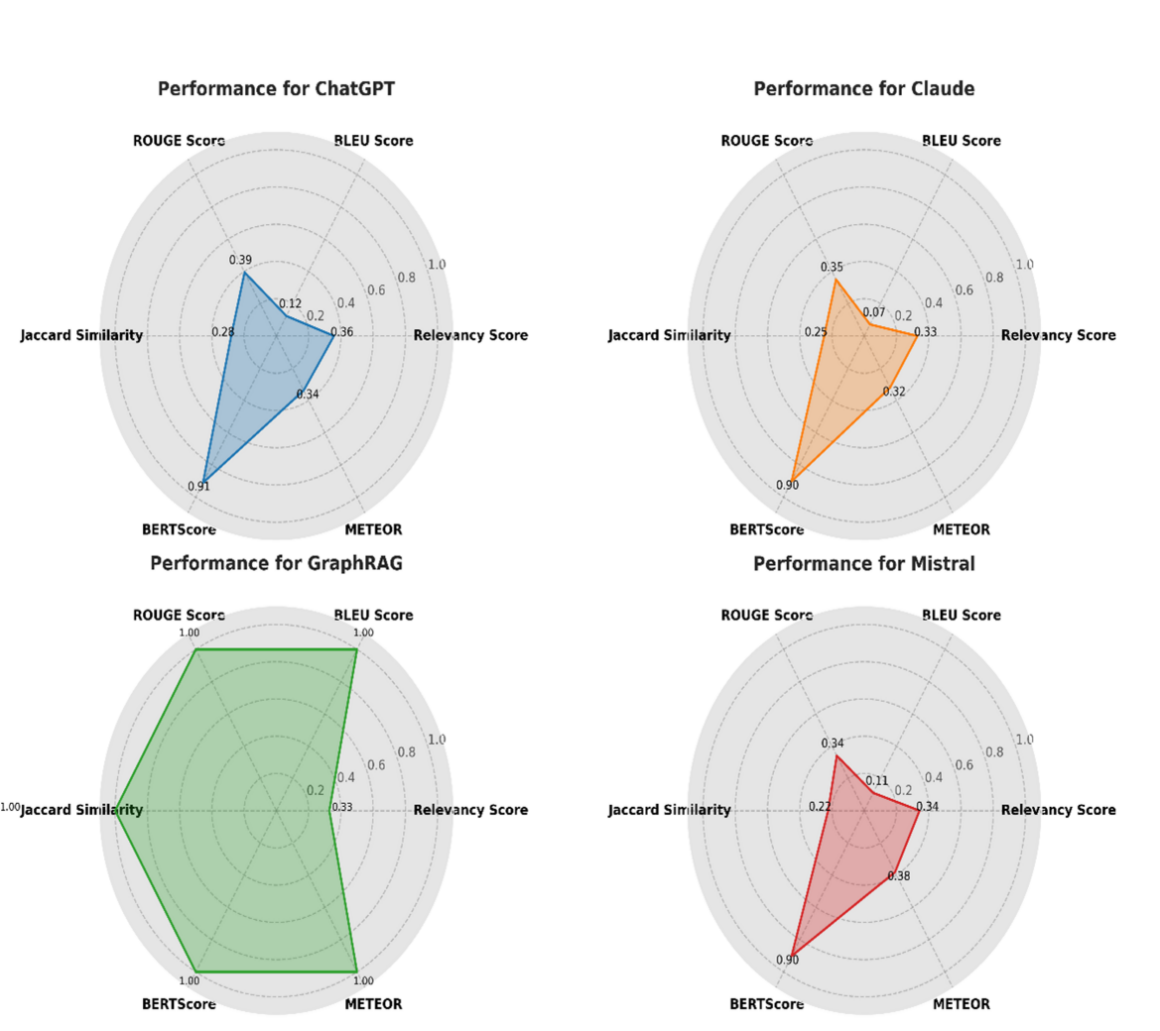
Radar chart visualizing model performance across key metrics. BLEU: bilingual evaluation understudy.

Figure 11 shows a radar chart (also known as a Kaviat diagram) of the same results, highlighting GraphRAG’s balanced strengths across multiple evaluation dimensions.

### Key Observations

1. Relevance Score: GraphRAG and BioMistral showed comparable results, aligning well with the clinical intent of queries.
2. BLEU Score: GraphRAG outperformed all other models, reflecting precise replication of validated clinical expressions.
3. Jaccard Similarity: GraphRAG highlighted superior consistency in medical terminology usage across responses.
4. BERTScore: The model achieved the highest semantic similarity, indicating deep contextual understanding.
5. METEOR: GraphRAG generated coherent and fluent responses suitable for clinical communication, comparable with ChatGPT and Claude.

These findings demonstrate the technical feasibility of the proposed GraphRAG-enabled local LLM. However, we stress that as a PoC evaluated in a simulated environment, the prototype is not ready to be deployed in real-world clinical settings. Even so, these results show that the GraphRAG approach effectively balances linguistic precision, contextual depth, and clinical relevance in GDM decision support scenarios. Besides BioMistral, ChatGPT, and Claude, new open-source LLMs such as LLaMA 3, Mistral 7B, and Phi-3 are becoming prevalent in health care AI. Although hardware and framework limitations prevented their inclusion in this study, we recognize their importance as baselines. Future work will add these models to expand our comparative analysis.

## Discussion

### Principal Findings

This study demonstrates that the GraphRAG-enabled local LLM consistently produces clinically relevant, contextually grounded, and medically precise responses for managing GDM. Through a rigorous benchmarking process against established open-source models, BioMistral, ChatGPT, and Claude, the GraphRAG approach achieved top-tier performance across all key evaluation metrics: BLEU Score, Jaccard Similarity, and BERTScore. The radar chart (Figure 11) illustrates well-balanced strengths of the model across multiple evaluation dimensions. Notably, first, GraphRAG matched BioMistral in Relevance Score, indicating strong alignment with the clinical intent behind queries of the health care providers. This score reflects how accurately the system understands and responds to the specific clinical context of the user, which is critical in decision support. Second, GraphRAG outperformed all models in BLEU Score and Jaccard Similarity, showcasing its ability to reproduce clinical phrasing with syntactic accuracy and maintain consistency in key medical terminologies, a vital factor for preserving the meaning of technical medical advice. Third, it achieved a superior BERTScore, showing deep semantic understanding. This reflects the capacity of the model to generate responses that not only match expected language structures but also accurately convey complex clinical relationships within GDM care. Finally, the superior performance across these diverse metrics stems from the architectural design of GraphRAG. By integrating domain-specific KGs with RAG, the system grounds its responses in verified clinical evidence rather than relying solely on probabilistic language patterns. This integration mitigates common challenges of general-purpose LLMs, such as hallucinations and domain irrelevance, ensuring that responses are both medically accurate and context-aware.

These findings validate the core hypothesis that combining structured medical knowledge and patient-specific information with GenAI significantly enhances the quality, safety, and reliability of AI-generated clinical recommendations.

### Implications for AI-Driven Clinical Decision Support

The empirical results of this study highlight the transformative potential of GraphRAG-powered local LLMs in advancing AI-enabled clinical decision support systems. This is particularly the case within specialized domains, such as GDM management, where expertise is scarce and uneven. By combining structured retrieval from a domain-specific KG with contextual generation, the system addresses the longstanding gap between unstructured clinical queries and structured evidence-based medical knowledge.

The GraphRAG approach addresses a critical limitation of general-purpose LLMs, such as ChatGPT and Claude, which often prioritize linguistic fluency over clinical accuracy. While these models can generate coherent responses, they frequently lack the domain specificity needed for accurate clinical guidance. In comparison, the responses of GraphRAG consistently align with established clinical guidelines, reflecting a deep understanding of current medical standards and practices. For example, when prompted to hear about GDM diagnosis, GraphRAG accurately recommended the glucose challenge and tolerance tests, mirroring clinical best practices. This indicates that the system is not merely generating plausible text but retrieving and contextualizing domain-specific evidence to support clinical decision-making.

The clinical utility of such contextually enriched responses is profound. In healthcare, where treatment decisions directly affect patient safety and outcomes, factual accuracy and contextual relevance are not optional but essential. The ability of GraphRAG to consistently deliver these qualities positions it as a valuable tool for supporting health care providers, particularly in low-resource or high-pressure clinical environments where access to specialist knowledge may be limited.

The practical utility of GraphRAG is further illustrated in Textbox 2, which presents a representative response to a clinically relevant query about managing insulin resistance in patients with GDM. Unlike generic language models, GraphRAG provides structured, evidence-aligned recommendations grounded in recent clinical guidelines.

Textbox 2. **GraphRAG response to a clinical query on managing GDM.**

*Query: What are the latest recommendations for managing insulin resistance in GDM patients?*

GraphRAG response: Recent studies suggest a multi-faceted approach to managing insulin resistance in GDM patients, including:

1. Personalized low glycemic index diets, emphasizing complex carbohydrates and fiber-rich foods.

2. Structured exercise programs combining aerobic and resistance training for at least 150 minutes per week.

3. Early initiation of metformin where lifestyle modifications are insufficient, as per the 2023 guidelines.

4. Continuous glucose monitoring to optimize insulin dosing and adjust lifestyle recommendations.

5. Regular screening for comorbidities such as hypertension and dyslipidemia, which contribute to insulin resistance.

The above sections highlight the ability of GraphRAG to transform unstructured clinical questions into actionable, guideline-compliant insights. By synthesizing evidence from domain-specific KGs, the system avoids unsupported claims and produces responses aligned with best clinical practices, supporting its role as a trustworthy clinical decision support tool.

### Contributions to AI in Health Care

This study advances the field of health care AI by presenting a scalable, contextually enriched clinical support system specifically designed for GDM management. We believe that our key contribution lies in the system’s ability to empower GPs and nonspecialist clinicians, particularly in underserved and resource-limited health care environments with limited access to endocrinology specialists and up-to-date clinical knowledge. By using a KG-driven retrieval process, the system surfaces context-specific clinical insights without requiring clinicians to conduct exhaustive manual literature reviews or consult multiple sources. Here, a word of caution is in order. We reiterate that the PoC works best as a clinical assistant; that is, a health practitioner must be in the loop. This is important given the dangers of unsupervised AI agents, which may usurp the role of a human caregiver without human oversight [44]. It is concerning that a recent, peer-reviewed (and in our view, misguided) study actually normalizes a doctor versus machine “Turing-test of authenticity” [45].

Furthermore, this study shows domain-specific superiority over general-purpose LLMs. While models such as ChatGPT and Claude can produce coherent responses, they lack the fine-tuned contextual sensitivity and clinical precision essential for specialized health care domains. In comparison, the architecture of GraphRAG is optimized to capture the complex relationships inherent in GDM management, such as patient history with risk factors, availability of interventions, and outcome pathways for follow-up medical care, thereby enhancing both response accuracy and clinical applicability.

This study contributes to a novel retrieval-augmented GenAI architecture that translates domain-specific medical knowledge into clinically actionable insights. It serves a need; namely, access to the latest, credible medical research in time- and resource-constrained environments. In health care, timely and science-based interventions are crucial.

### Technical Innovations Driving Performance Gains

The robust performance of the GraphRAG-enabled local LLM stems from the integration of 3 core technical innovations that address longstanding limitations in clinical AI systems.

First, the KG integration allows for the structured representation of complex clinical relationships between risk factors, interventions, symptoms, and outcomes. Unlike flat text embedding, the KG enables the system to reason over interconnected entities and contextual dependencies, ensuring that recommendations are grounded in the complete clinical scenario rather than isolated data points.

Second, the RAG framework of the system addresses the gap between static model knowledge and dynamic, evolving medical evidence. The system mitigates temporal gaps by integrating retrieval from an up-to-date domain-specific KG. It reduces the risk of hallucinated or outdated responses, a common flaw in general-purpose LLMs trained on static corpora.

Third, the domain-specific adaptation of the model through targeted prompting strategies and fine-tuning on GDM-related interventions enhances its ability to understand and accurately apply specialized clinical terminology in localized contexts. This adaptation ensures that the system’s responses reflect the nuanced requirements of GDM management, capturing both the syntactic precision and semantic depth necessary for high-stakes clinical situations like emergency room triage.

We believe that these innovations enable the system to move beyond generic language generation, delivering interpretable, actionable, and clinically validated responses. This advancement represents a meaningful step toward reliable AI-assisted clinical decision-making, especially for chronic disease management scenarios where timely and context-aware recommendations are essential.

### Conclusions

#### Limitations and Challenges for Clinical Deployment

While the initial results from this PoC study are promising, several critical limitations must be addressed before GraphRAG can be translated into clinical practice. Intended as a PoC, the system has not undergone field validation. Future studies involving real-world patient interactions, clinician feedback, and longitudinal follow-up are essential to establish the model’s safety, reliability, and usability in live health care environments.

A second major consideration concerns data privacy and protection. Although this PoC did not involve patient-level data, real-world deployments would necessitate strict adherence to data protection frameworks. The integration of privacy-preserving learning paradigms, such as federated learning, would allow models to be trained on decentralized clinical data without exposing sensitive patient information. Complementary techniques, such as blockchain for differential privacy and secure multiparty computation, could further protect patient confidentiality.

The interpretability of AI-generated clinical responses remains a pressing challenge. While GraphRAG uses structured retrieval to enhance contextual grounding, clinicians must be able to trust and explain its outputs. Future iterations of the system should integrate explainability frameworks such as Shapley Additive Explanations or Local Interpretable Model-agnostic Explanations, enabling clinicians to trace and retrieve evidence on how specific KG pathways contribute to a given clinical recommendation.

In addition, seamless workflow integration will be critical for adoption. Clinical decision support systems must embed naturally within existing electronic health record platforms, minimizing disruption to physician workflows. Without such integration, even the most accurate systems risk being underused in clinical practice.

As with many multistage AI pipelines, GraphRAG is also subject to the risk of error propagation, where inaccuracies in earlier stages, such as entity extraction or graph construction, may be compounded in downstream response generation. While our current prompt engineering and domain-specific graph design reduce this risk, future versions will integrate intermediate validation checkpoints, feedback loops, and retrieval-failure auditing to ensure response fidelity and system transparency.

Another key limitation is the reliance on English-language peer-reviewed articles from a single aggregator (Semantic Scholar). This has excluded regional or non-English medical literature with culturally adapted GDM interventions. Future work should incorporate multilingual and regionally diverse corpora to improve the model’s generalizability and contextual sensitivity, particularly in Global South health care settings.

Finally, the computational demands of GraphRAG’s RAG architecture present scalability challenges. The latency and resource consumption must be optimized to support real-time inference in time-sensitive clinical settings, especially in environments where computational capacity may be limited. Addressing these challenges is essential for transitioning GraphRAG from an academic PoC to a clinically viable, ethically responsible AI system.

#### Broader Implications and Future Research Directions

Building on the demonstrated feasibility of our PoC, our future research agenda is designed to advance the GraphRAG framework along 2 primary axes: strategic domain expansion and core technical refinement. First, we propose to strategically adapt the framework for other data-intensive clinical areas, including cardiovascular disease, oncology, and mental health, where evidence-grounded decision support is crucial. Second, we will enhance the core retrieval engine by integrating advanced algorithms, such as contextual BM25 and embedding-based summarization, to improve precision. To improve robustness and transparency, we propose implementing new retrieval-specific metrics, such as recall and failure rates. We have established a roadmap and aim to pursue these enhancements in our next research cycle, solidifying the GraphRAG pipeline as a viable tool for real-world clinical decision support.

The legal, ethical, and intellectual property considerations will also shape future deployments. To ensure transparency and reduce legal risks, future iterations will prioritize training on open-access datasets such as PubMed Central, adhering to responsible AI development practices and open science principles.

To protect patient privacy and mitigate algorithmic bias will remain core ethical imperatives. The federated learning and anonymized blockchain solutions could support decentralized training across institutions without compromising patient confidentiality. Bias audits, fairness-aware modeling, and hallucination mitigation strategies, such as reranking retrieved evidence and diversifying training datasets, will improve the reliability and equity of the system’s clinical recommendations. In such a trusted platform, integrating GraphRAG with real-time patient data could enable personalized clinical decision support, customizing recommendations to individual genetic profiles, lifestyle factors, and environmental exposures. This evolution toward precision medicine would represent a significant leap forward in AI-driven health care delivery.

To overcome the limitation of computational costs, the enhanced system will require architectural optimizations to enable scalability in resource-constrained clinical settings. Techniques such as prompt caching, adaptive chunking of graph queries, and hybrid retrieval strategies will reduce computational costs and response latency. This will support deployments in low-bandwidth environments, such as rural clinics and community health centers.

In the long term, retrieval-augmented LLMs, such as GraphRAG, are envisioned not as autonomous clinical agents but as clinical copilots, supporting, rather than replacing human clinicians. Their evaluation in live clinical workflows will be critical to determining their optimal role as decision-support systems. A reflective perspective on this motivation is presented in Textbox 3, showing the personal origins of our research question.

Textbox 3. **Closing vignette on gestational diabetes.**

*“I do not wish to alarm you, Mrs. Sharma, but you have been diagnosed with gestational diabetes and your baby is 10 pounds at birth. Both of you need to be careful.”*

[Ward Nurse in Singapore’s Kandang Kerbau Maternity Hospital to the mother of the last author, circa 1961]

In 2022, the mother passed away peacefully at the age of 88, her diabetes controlled with insulin injections for decades. The “baby” (the last author and principal investigator of this study [RS]) was diagnosed with type 2 diabetes at the age of 60, giving rise to our research question of whether a graph-based retrieval-augmented generation solution could change the outcome for both with timely, relevant best practices.

In closing, this paper sought to establish the feasibility of a GraphRAG-enabled local LLM architecture for generating clinically relevant, context-aware responses in the management of diseases, such as GDM [46]. By integrating domain-specific KGs with RAG, the system outperformed general-purpose LLMs across multiple evaluation metrics, offering evidence-grounded and terminologically precise clinical recommendations. While this work serves as a technical PoC, future research will need to focus on (1) prospective clinical validation involving real-time patient interaction, (2) multimodal agents to improve accessibility and cultural sensitivity, and (3) integration of explainable AI modules, such as Shapley Additive Explanations–based KG traceability, resulting in enhanced trust and transparency for the 2 key humans in the loop – the patient and her doctor. Ultimately, we believe the transformative potential of AI-powered decision support tools will personalize care and improve clinical outcomes, particularly in underserved societies.

## Abbreviations

AI: artificial intelligence
BLEU: bilingual evaluation understudy
GDM: gestational diabetes mellitus
GenAI: generative artificial intelligence
GP: general practitioner
GraphRAG: graph-based retrieval-augmented generation
KG: knowledge graph
LLM: large language model
ML: machine learning
PoC: proof-of-concept
PRISMA: Preferred Reporting Items for Systematic Reviews and Meta-Analyses
RAG: retrieval-augmented generation
SML: small language model
T2D: type 2 diabetes
